# The Association Between Occupation, Attitudes Towards Mental Health Problems in the Workplace and Mental Health Stigma

**DOI:** 10.1192/j.eurpsy.2025.392

**Published:** 2025-08-26

**Authors:** G. Cheung, A. Ronaldson, C. Henderson

**Affiliations:** 1 Hull York Medical School; 2 York and Scarborough Teaching Hospitals NHS Foundation Trust, York; 3 Centre for Implementation Science, Health Service and Population Research Centre, Institute of Psychiatry, Psychology, & Neuroscience, King’s College London, London, United Kingdom

## Abstract

**Introduction:**

Although mental health problems are common in the workplace, discrimination against mental health is still prominent. Characteristics of one’s own occupation can influence aspects of stigma and attitudes to mental health problems in the workplace context, but there are limited studies examining differences in them between occupational groups.

**Objectives:**

We investigated occupational differences in mental health stigma, and attitudes to both mental and physical health in the workplace.

**Methods:**

Data from the British Social Attitudes 2015 survey were used. Logistic regression models were conducted to investigate associations between occupational categories and stigma as measured by desire for social distance, as well as attitudes towards mental and physical health in the workplace. Occupational categories were based on the National Statistics Socio-economic Classification. Measures for attitudes to mental and physical health in the workplace include whether paid work is good for mental and physical health, how soon one should return to work following depression, whether work can help speed recovery from depression, whether one’s medical history should affect their promotion, including depression, schizophrenia and diabetes. Desire for social distance from people with depression and schizophrenia was measures using unlabelled vignettes.

**Results:**

We found occupational differences in attitudes towards mental and physical health in the workplace, but not in levels of stigma. People in lower supervisory and technical (group 4), semi-routine and routine (group 5) occupations were more likely to have negative attitudes towards mental and physical health in the workplace compared to managerial and professional occupations (group 1). Both occupation groups were less likely than group 1 to believe that paid work is good for mental health (group 4: odds ratio (OR) = 0.38, 95% confidence interval (CI) = 0.24-0.61; group 5: OR = 0.34, 95% CI = 0.24-0.49). They were also less likely to believe that people with depression should return to work when they can do some or most of the job (group 4: OR = 0.67, 95% CI = 0.48-0.94; group 5: OR = 0.52, 95% CI = 0.41-0.66). People in group 5 were less likely to believe that paid work is good for physical health (OR = 0.69, 95% CI = 0.53 to 0.89) and that having schizophrenia should not affect promotion at work (OR = 0.78, 95% CI = 0.62-0.97) than group 1.

**Conclusions:**

Our study reinforced the importance of job characteristics on attitudes to mental health in the workplace. First, employers need to invest more in improving their employees’ well-being. Second, governments should provide more support and resources for small companies to help them develop mental health policies and practices. Third, modifications need to be made to improve job control for employees and to ensure enough reasonable adjustments can be made.

**Disclosure of Interest:**

G. Cheung: None Declared, A. Ronaldson: None Declared, C. Henderson Consultant of: C.H. has received consulting fees from Lundbeck and educational speaker fees from Janssen.

